# Siglec-F-dependent negative regulation of allergen-induced eosinophilia depends critically on the experimental model

**DOI:** 10.1016/j.imlet.2014.03.008

**Published:** 2014-07

**Authors:** Sarah J. McMillan, Hannah E. Richards, Paul R. Crocker

**Affiliations:** Division of Cell Signalling & Immunology, College of Life Sciences, University of Dundee, Dundee, Scotland, UK, DD1 5EH

**Keywords:** Siglec, sialic acid binding immunoglobulin like lectin, OVA, ovalbumin, WT, wild type, KO, knock out, KI, knock in, ITIM, immunoreceptor tyrosine based inhibitory motifs, Siglec-F, Siglec-8, Eosinophil, Allergic airway inflammation

## Abstract

Siglec-8 and siglec-F are paralogous membrane proteins expressed on human and murine eosinophils respectively. They bind similar sialylated and sulphated glycans and mediate eosinophil apoptosis when cross-linked with antibodies or glycan ligands. In models of allergic eosinophilic airway inflammation, siglec-F was shown previously to be important for negatively regulating eosinophilia. It was proposed that this was due to siglec-F-dependent apoptosis, triggered *via* engagement with ligands that are upregulated on bronchial epithelium. Our aim was to further investigate the functions of siglec-F by comparing two commonly used models of ovalbumin-induced airway inflammation that differ in the dose and route of administration of ovalbumin. In confirmation of published results, siglec-F-deficient mice had enhanced lung tissue eosinophilia in response to intranasal ovalbumin delivered every other day. However, following aerosolised ovalbumin delivered daily, there was no influence of siglec-F deficiency on lung eosinophilia. Expression of siglec-F ligands in lung tissues was similar in both models of allergen induced inflammation. These data demonstrate that siglec-F-dependent regulation of eosinophilia is subtle and depends critically on the model used. The findings also indicate that mechanisms other than ligand-induced apoptosis may be important in siglec-F-dependent suppression of eosinophilia.

## Highlights

• Siglec-F-dependent negative regulation of eosinophilia depends on experimental model.
• Siglec-F-dependent suppression of lung eosinophilia may not depend on ligand-induced apoptosis.
• Implications for therapeutic approaches to treating human disease in which siglec-8, is targeted.

## 1 Introduction

Siglecs-8 and -F are paralogous membrane proteins highly expressed on human and mouse eosinophils respectively. They are members of the CD33-related sialic acid binding Ig-like lectin (siglec) family and contain immunoreceptor tyrosine based inhibitory motifs (ITIM) and ITIM-like motifs in their cytoplasmic tails that are implicated in negative regulatory functions [1]. Previous studies have shown that cross-linking of siglecs-8 and -F *in vitro* using specific antibodies or glycan ligands can lead to eosinophil apoptosis [2,3]. This has raised the possibility that selective targeting of these cells in diseases such as allergic asthma could be beneficial [2–5]. Although murine siglec-F is not orthologous with siglec-8, their similarities in expression pattern [6,7], proapoptotic properties [4,8], and ligand binding preference [6] suggest that these proteins play equivalent functional roles. Therefore, analysis of siglec-F-deficient mice is likely to give insights into siglec-8 functions in humans.

Siglec-F has been shown to act as a negative regulator of ovalbumin (OVA)-induced eosinophilia since OVA-primed and challenged siglec-F-deficient mice had enhanced eosinophilic airway inflammation and increased numbers of eosinophils in the blood and bone marrow [9,10]. Moreover, siglec-F antibodies were shown to reduce eosinophilic inflammation in a model of OVA-induced airway inflammation [11]. Siglec-F ligands are expressed on bronchial epithelium and inflammatory leukocytes, including eosinophils themselves [9,10,12]. Two recent studies demonstrated that the sialyltransferase ST3Gal-III is required for siglec-F ligand expression and that ST3Gal-III-deficient mice exhibited increased lung eosinophilia in a model of OVA-induced allergic lung inflammation [13,14]. Collectively, these data have given rise to the hypothesis that engagement of siglec-8 or siglec-F with sialylated ligands leads to eosinophil apoptosis and negative regulation of eosinophil inflammation in the lung.

The above studies on siglec-F-dependent modulation of eosinophilia have been performed using a model of OVA-induced airway inflammation in which OVA-primed mice are challenged intranasally with OVA. Since it has been well documented that use of different murine models of allergic airway disease can result in contrasting data [15,16], we sought to compare the development of OVA-induced airway eosinophilia using two different models with previously unreported siglec-F-deficient mice generated in our laboratory.

Here we demonstrate that, in contrast to intranasal OVA delivery every other day, when aerosolised OVA was delivered daily, the absence of siglec-F did not lead to increased numbers of lung eosinophils. These differences in siglec-F-dependent eosinophil recruitment could not be explained by altered siglec-F ligand expression in the lung which was similar in both models. Our findings suggest that the negative regulatory effects of siglec-F may be overridden under certain inflammatory conditions and have important implications for the translation of siglec-F in mouse models of allergic inflammation to human siglec-8 in asthma.

## 2 Materials and methods

### 2.1 Generation and characterization of siglec-F-deficient mice

**Fig. 1:**
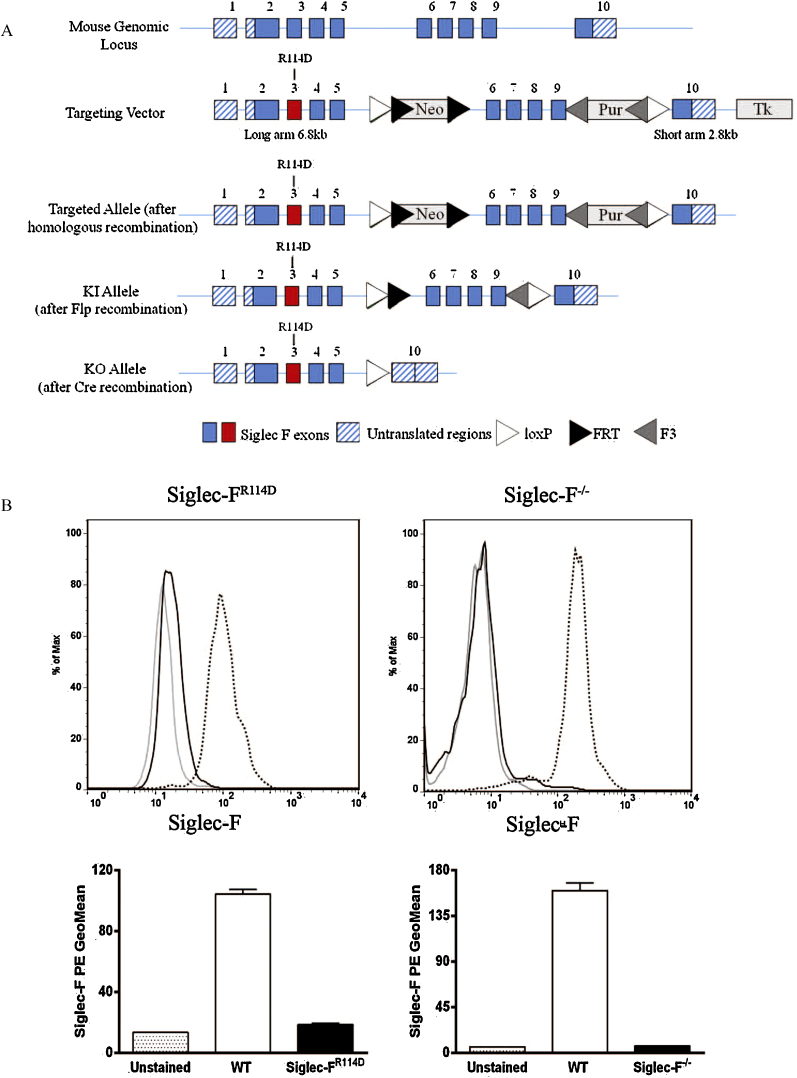
Generation and characterization of siglec-F-deficient mice. (A) Schematic representation of *Siglec-F* locus, the gene targeting vector to introduce R114D mutation in exon 3 (that contains the sialic acid binding site) and the targeted allele, with the aim of generating siglec-F^R114D^ ‘knock-in’ (KI) mice expressing full length protein that lacks the ability to bind sialic acid. The ‘knock-out’ (KO) allele was generated following a cross of siglec-F^R114D^ mice with Bal1 cre mice to excise exons 6–9, in order to generate siglec-F^−/−^ mice (Neo, neomycin; Pur, puromycin; Tk, thymidine kinase; FRT, neomycin resistance site; F3, puromycin resistance site). (B) Flow cytometric analysis of siglec-F on blood eosinophils (determined by SSc/Gr1^mid^ expression) from WT, siglec-F^R114D^ and siglec-F^−/−^ mice. Top panels show representative histograms of siglec-F staining from unstained (gray line) and WT (dotted line) and siglec-F deficient (black line) mice. Lower panels present mean ± S.D. GeoMean of siglec-F fluorescence from 2 to 4 mice per group.

Siglec-F^R114D^ ‘knock in’ (KI) mice, carrying an Arg114 to Asp mutation in the siglec-F gene predicted to abolish sialic acid binding [10], were generated by Taconic Artemis using C57BL/6 ES cells and the targeting strategy illustrated in Fig. 1A. Further crossing of homozygous siglec-F^R114D^ mice with Tg (Nes-cre)1Wme/J (Bal1 cre) mice produced offspring with mosaicism/partial deletion of exons 6–9 in adult organs (including germline), resulting in the truncated siglec-F ‘knock out’ (KO) allele shown in Fig. 1A. Mosaic offspring were then used to generate siglec-F^−/−^ mice, which was confirmed by PCR. The following primers gave rise to a wild-type product (202 bp), a KI product (321 bp) or a KO product (363 bp); 1928_35; CCTGATGTCATGTGTGAAGTCG, 1928_36; GATTTCAGGCGTGTGATTGC, 1928_38; CTCCTGAGGGCTGAGACTATAGG. WT, siglec-F^R114D^ and siglec-F^−/−^ mice were generated using parents from heterozygous intercrosses on C57BL/6 background. Mice were viable and fertile and no abnormalities were found in baseline total blood cell counts (data not shown). Mice were bred and maintained under specific pathogen-free conditions at the University of Dundee. All procedures were carried out with institutional ethics approval, under home office license and were performed in accordance with the UK 1986 Animals (Scientific Procedures) Act. Siglec-F expression was assessed by flow cytometry of eosinophils in blood, bone marrow and lung tissue digest from WT and siglec-F deficient mice using both a rat anti-siglec-F mAb (Clone E50-2440, BD Biosciences, Oxford, UK) and a sheep anti-siglec-F pAb [8]. Eosinophils were delineated with anti-Gr-1 antibody (RB6-8C5; eBioscience, Hatfield, UK) and side-scatter properties. Fc receptor binding was blocked with 2.4G2 (hybridoma) prior to surface staining. Following staining, cells were fixed in 1% formaldehyde. Data were collected using a LSR Fortessa (BD Biosciences) and analyzed using Flowjo 7.5 software (Treestar).

### 2.2 Induction of allergic airway inflammation

**Fig. 2:**
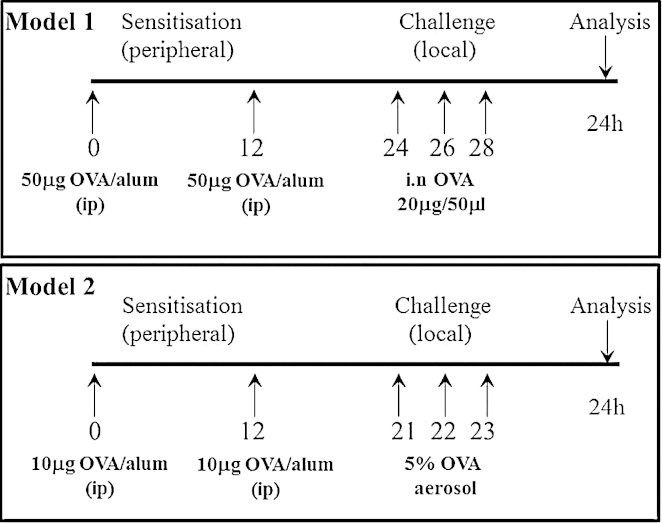
Schematic of the two models of OVA-induced airway inflammation tested in siglec-F deficient mice.

Allergic airway inflammation was induced in groups of 8-week old female siglec-F-deficient mice and their WT littermates using two models (Fig. 2). Mice were sensitized on days 0 and 12 using intraperitoneal OVA (Chicken egg, Grade V, Sigma, Poole, UK), at a concentration of 50 μg/mouse (model 1) in 0.2 ml alum (Alu-Gel-S; Serva, AMS Biotechnology, Abingdon, UK), or 10 μg/mouse in 0.2 ml alum (model 2). To induce local inflammation to the airways for model 1, mice were anaesthetized using isofluorane and 20 μg in 50 μl OVA instilled into the nostrils on days 24, 26 and 28 [10]. For model 2, mice were challenged daily with 5% OVA (aerosolized for 20 min) *via* the airways between days 21 and 23. For both models, mice were sacrificed by exsanguination under terminal anesthesia (ketamine, 100 mg/kg/xylazine, 10 mg/kg, i.p.) 24 h after the final OVA administration and lung tissue inflammation assessed.

### 2.3 Quantification of lung cellular inflammation

Lung inflammation was assessed in collagenase digested lung tissue as previously described [17]. Differential cell counts of lung tissue digests (40× magnification; total area 0.5 mm^2^ per area randomly selected) were performed on Diff Quik stained cytospins. All differential counts were performed blind and in a randomized order at the end of the study by the same observer.

### 2.4 Siglec-F ligand expression

Siglec-F ligand expression was assessed as described [12], with minor modifications. 8 μm sections were blocked for 1 h with 5% normal sera from mouse and goat (Sigma) diluted in 0.5% casein solution (Vector Labs, Peterborough, UK). 1 μg/ml siglec-F-Fc (in house) was pre-complexed to anti-human Fc Alexa-488 (Jackson ImmunoResearch, Newmarket, UK) at a ratio of 1:1 for 1 h 4 °C prior to incubation on slides for 24 h 4 °C. To evaluate background fluorescence, sections were stained with an irrelevant Fc-protein. To evaluate sialic acid dependent binding, sections were treated with 0.17 U/mL *Vibrio cholerae* sialidase (Sigma) in HBSS at 37 °C for 1 h prior to block. All sections were incubated with DAPI (Vector Labs) at 0.5 μg/mL in PBS for 5 min to label nuclei and confocal images were acquired (LSM 700 microscope and Zen 2009 software; Carl Zeiss). Multi-channel images were created and processed in parallel using Photoshop software (Adobe).

### 2.5 Data analysis

Statistics were performed using GraphPad Prism 6 software (GraphPad Software, La Jolla, USA) and significance between groups was tested using a Mann Whitney *U* test. A *p* value of less than or equal to 0.05 was considered significant.

## 3 Results

### 3.1 Generation of siglec-F deficient mice

In the course of attempting to generate siglec-F^R114D^ ‘KI’ mice carrying a mutation in the sialic acid binding site, we found that the siglec-F expression was barely detectable on blood eosinophils from siglec-F^R114D^ mice by flow cytometry (Fig. 1B). This was not due to loss of the epitope recognized by the anti-siglec-F mAb since similar low staining of siglec-F^R114D^ eosinophils was observed with an anti-siglec-F polyclonal antibody known to recognize more than one epitope (data not shown). The greatly reduced siglec-F expression in siglec-F^R114D^ mice was most likely due to disruption of gene transcription arising from the targeting strategy used, since quantitative PCR analysis showed that siglec-F mRNA in bone marrow from siglec-F^R114D^ mice was decreased to ∼35% of levels found in WT mice (data not shown). We also generated siglec-F^−/−^ KO mice by crossing siglec-F^R114D^ mice with bal-cre mice (Fig. 1A). As expected, lack of eosinophil siglec-F expression was observed in the blood (Fig. 1B), bone marrow and lung tissue digest of these mice in comparison with WT mice (data not shown). The percentage of eosinophils in the blood and bone marrow of WT and siglec-F^−/−^ mice were comparable at baseline (Blood: WT, 1.7% ± 0.4; siglec-F^−/−^, 1.4% ± 0.2; Bone marrow: WT, 3.7% ± 0.3; siglec-F^−/−^, 3.6% ± 0.1).

### 3.2 Investigating the functional role for siglec-F *in vivo*

**Fig. 3:**
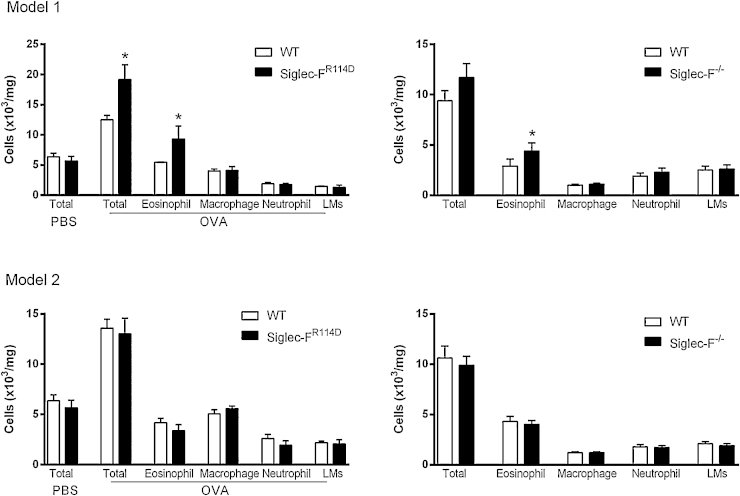
Siglec-F-dependent negative regulation of allergen-induced eosinophilia depends critically on the experimental model used. WT, siglec-F^R114D^ and siglec-F^−/−^ mice were subjected to allergic inflammation using either model 1 or model 2. Total and differential cells counts were enumerated in Diff Quik stained cytospins of collagenase digests of lung tissue (LMs; lymphomononuclear cells). Data are expressed as mean ± S.E.M., *n* = 4–6/group, *p ≤ 0.05 using Mann Whitney *U* Test.

To extend previous work using a single model of OVA-induced airway inflammation [10], we compared two commonly used models in the siglec-F^R114D^ and siglec-F^−/−^ mice. Models 1 and 2 differ in the concentration of OVA used for sensitization and in the frequency and concentration of OVA challenge to induce local inflammation in the lung (Fig. 2). Similar to the total cell counts (Fig. 3), lung eosinophil numbers did not differ between WT and siglec-F^−/−^ mice at baseline in the absence of OVA (means ± S.E.M., WT: 0.5 × 10^3^ mg^−1^ ± 0.06; siglec-F^−/−^: 0.5 × 10^3^ mg^−1^ ± 0.05). Using model 1, it was previously shown that mice deficient in siglec-F had enhanced eosinophilia induced by OVA [10]. Indeed, we confirmed this phenotype in lung tissue digest using the siglec-F^R114D^ and siglec-F^−/−^ mice reported here (Fig. 3). However, using model 2, where the amount of allergen used for sensitization is lower and the dosing of local OVA to the airways is given daily rather than every other day, there was no influence of siglec-F deficiency on eosinophil recruitment to the lung tissue, using either siglec-F^R114D^ or siglec-F^−/−^ mice (Fig. 3).

### 3.3 Siglec-F ligand expression *in vivo*

**Fig. 4:**
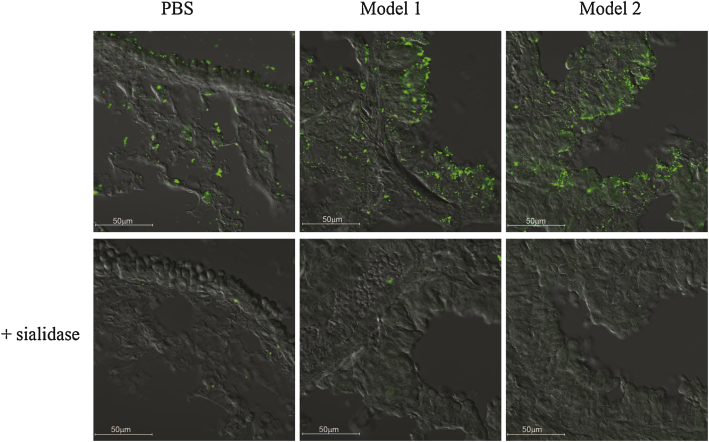
Comparable expression of siglec-F ligands in the lung tissue of model 1 and model 2 induced allergic airway inflammation. The expression of siglec-F ligands in lung tissue taken from PBS or OVA-treated WT mice was measured using pre-complexes of siglec-F-Fc/anti-Fc. Sections shown in lower panels were treated with sialidase prior to staining. Sections were analyzed by confocal microscopy (LSM 700 microscope and Zen 2009 software; Carl Zeiss) and images collected with an α-Plan-Apochromat 40× NA 1.46 objective. Scale bars represent 50 μm.

In order to determine if the difference in siglec-F-dependent suppression of lung eosinophilia for the two models was linked to differential expression of siglec-F ligands, lung tissue sections from WT mice were stained with siglec-F-Fc (Fig. 4). As reported previously [12], in untreated mice siglec-F-Fc staining was observed both in the apical surface of airway epithelial cells where high levels of mucins are present, as well as in cells in the alveolar bed. This staining was increased when mice were exposed to OVA and was also seen on inflammatory cells. However, the pattern of staining and intensity did not differ between the two models of OVA-induced airway inflammation (Fig. 4). Staining was sialic acid-dependent and specific since sialidase treatment greatly reduced the signals (Fig. 4) and no staining was observed using an irrelevant Fc-protein (data not shown).

## 4 Discussion

Using two well-characterized models of OVA-induced airway inflammation that differ in the amount of OVA used for sensitization and in the route/concentration of OVA delivered locally to the lung, we demonstrate that the previously reported effect of siglec-F on suppression of eosinophilia was only observed in one of the models in which OVA was instilled intranasally every other day [10]. In contrast, when OVA was aerosolised daily, the negative regulatory effects of siglec-F were not observed. In view of current thinking that siglec-F-dependent negative regulation of lung eosinophilia is due to siglec-F interactions with sialylated lung ligands triggering eosinophil cell death [18], it was important to compare ligand expression in both models. However, no obvious differences were observed. These data therefore raise a number of questions regarding the *in vivo* inhibitory functions of siglec-F that seem to critically depend on subtle features of the inflammatory environment.

Rather than regulating eosinophil numbers *via* ligand-induced apoptosis, siglec-F may directly suppress eosinopoiesis under the inflammatory conditions of model 1. Consistent with this notion, siglec-F-deficient mice subjected to model 1 exhibited exaggerated eosinophilia in the bone marrow and blood, as well as increased eosinophil precursors in the bone marrow [10]. Similar to other CD33-related siglecs, siglec-F contains ITIM-like motifs known to recruit tyrosine phosphatases SHP-1 and SHP-2 [1]. A related ITIM-receptor, CEACAM-1 expressed on neutrophils was found to be a negative regulator of granulopoiesis *via* SHP-1 recruitment and inhibition of G-CSF/STAT3 signaling pathway [19]. Moreover, *via* association with SHP-1, crosslinking of IRp60/CD300a inhibited the activation of eosinophils in response to GM-CSF/IL-5 [20], so it is plausible that siglec-F in association with SHP-1/2, mediates a similar function in eosinopoiesis in response to IL-5 and GM-CSF signaling *in vivo* [21,22] despite not being required *in vitro* [23].

Another possibility is that the inhibitory effect of siglec-F on eosinophil numbers depends on the magnitude and kinetics of the inflammatory response. Although eosinophil recruitment was comparable between the two models, inhibitory signals from siglec-F observed in model 1 could be masked in model 2 due to the higher frequency of OVA challenge. Besides eosinophils, siglec-F is also expressed on alveolar macrophages [24], and is upregulated on *in vitro* activated CD4^+^ and CD8^+^ T cells [10]. It is well documented that allergen-induced airway inflammation is driven by CD4^+^ Th2 cells [25,26] that release cytokines such as IL-5 that promote eosinophil proliferation and survival [27]. Therefore, siglec-F-dependent suppression of Th2 cell cytokine production could also be a factor in suppression of eosinophil numbers in model 1, but could be lost in model 2 due to the increased frequency of antigenic stimulation. However, in our hands, although numbers of recruited CD3^+^CD4^+^ T cells were low, we could not detect expression of siglec-F on T cells in either model (data not shown). Expression of ITIM-containing CD33-related siglecs in macrophages has been shown to down-regulate cytokine production in response to inflammatory mediators [28,29]. Hence, if model 1 (but not model 2) leads to siglec-F-dependent suppression of alveolar macrophage-derived factors that promote eosinophilia, this could also contribute to the results observed in this study.

Taken together it is clear that delineating the role of siglec F in allergen induced airway inflammation is complex, with the choice of model system being crucial. Recently, models have been developed that negate the need for a peripheral sensitization step, use environmentally and clinically relevant allergens, and replicate the relevant features of the human disease [30,31]. Therefore, it will be important to study siglec-F deficient mice in such models to define the functional role *in vivo*. Moreover, cell-type specific deletion of siglec-F in eosinophils, alveolar macrophages and T cells could help dissect the relative contribution of each cell type to siglec-F-dependent suppression of eosinophilia.

## 5 Conclusion

In conclusion, we have shown that, depending on the nature of the allergy model used, the negative regulatory role of siglec-F on eosinophilia may be overridden. These findings have important implications for the translation of siglec-F in mouse models of allergic inflammation to human siglec-8 in asthma and suggest that further analysis of siglec-F mice in a number of model systems should be evaluated.

## Conflict of interest

The authors declare no conflict of interest.

## Authorship contribution statement

S.J.M. and P.R.C. designed the study and prepared the manuscript; S.J.M. and H.E.R. performed experiments and processed the data.
